# Applying Biomarkers in Treat-to-target Approach for IBD

**DOI:** 10.1007/s11894-025-00991-7

**Published:** 2025-06-20

**Authors:** Megan Lutz, Sara Horst, Freddy Caldera

**Affiliations:** 1 School of Medicine & Public Health, Department of Medicine, Division of Gastroenterology and Hepatology, University of Wisconsin, Madison, WI USA; 2 Division of Gastroenterology, Department of Medicine, Vanderbilt University Medical Center, Nashville, TN USA

**Keywords:** Biomarkers, Fecal calprotectin, C-reactive protein, Treat-to-target

## Abstract

**Purpose of review:**

The treatment of inflammatory bowel disease (IBD) has evolved significantly over time based on “treat-to-target”, an approach which uses sequential objective makers to monitor response to therapy with the ultimate goal of achieving endoscopic healing. Biomarkers, including C-reactive protein and fecal calprotectin, are an important noninvasive intermediate step in this treatment approach as well as in routine monitoring of disease activity. While widely utilized, there is significant variability and some uncertainty in biomarker implementation; this review summarizes evidence for the use of biomarkers in IBD.

**Recent findings:**

The Selecting Therapeutic Targets in Inflammatory Bowel Disease (STRIDE) update in combination with the 2023 American Gastroenterological Association (AGA) guidelines on the role of biomarkers in the management of both Crohn’s disease and ulcerative colitis have offered significant new guidance for those who manage IBD.

**Summary:**

Biomarkers offer important insight into disease activity and can be used to track progress toward deeper levels of remission in IBD.

## Introduction

Inflammatory bowel disease (IBD) including Crohn’s disease (CD) and ulcerative colitis (UC) can be progressive conditions which, when not treated promptly, may lead to irreversible damage and disability*.* To prevent these complications, management of IBD has evolved significantly over time, shifting from an approach guided by symptoms alone to one that proactively incorporates objective findings, tracking progress toward deeper levels of remission [1]. This so called “treat-to-target” approach, initially outlined by the Selecting Therapeutic Targets in Inflammatory Bowel Disease (STRIDE) working group in 2015 and later updated in 2021 as STRIDEII, now serves as an important treatment guide for IBD clinicians [2, 3]. Under this approach, sequential key benchmarks are used to assess treatment response and adjust therapy accordingly, beginning with a short-term goal of symptom improvement, progressing to an intermediate goal of biomarker normalization, and ultimately aiming for endoscopic remission. If, at any point these goals are not met, therapeutic adjustments can be made and patient response again closely monitored. Achieving endoscopic healing, the ultimate goal in the treat-to-target approach, has been linked with key outcomes in IBD. In CD, mucosal healing has been associated with sustained clinical response as well as decreased bowel damage, need for steroid treatment, and major abdominal surgery [4–6]. Similarly, in UC, endoscopic healing, commonly defined as a Mayo Endoscopic Score (MES) of 0–1, has been associated with decreased rates of hospitalization, need for steroid treatment, colectomy, dysplasia and colon cancer [6, 7].

Biomarkers, surrogate markers for disease activity including C-reactive protein (CRP) and fecal calprotectin (FC), have therefore become increasingly utilized noninvasive measures of IBD activity as an intermediate step in the treat-to-target approach. They provide distinct advantages over traditional assessment methods such as CT or colonoscopy as they can be assessed rapidly, followed serially, and avoid significant cost and patient burden. Their use has also been associated with greater likelihood of remission [8]. Biomarkers cannot replace endoscopy for disease assessment as they are only surrogates for disease activity, and their accuracy depends greatly on the clinical presentation including symptom severity. In addition, endoscopy will remain necessary for certain indications such as dysplasia detection and evaluation for cytomegalovirus. Nonetheless, biomarkers can be an important data point to follow in a patient’s disease course, especially in those who have a known history of biomarker elevation or known correlation of biomarkers with endoscopic activity [9, 10].

**Fig. 1:**
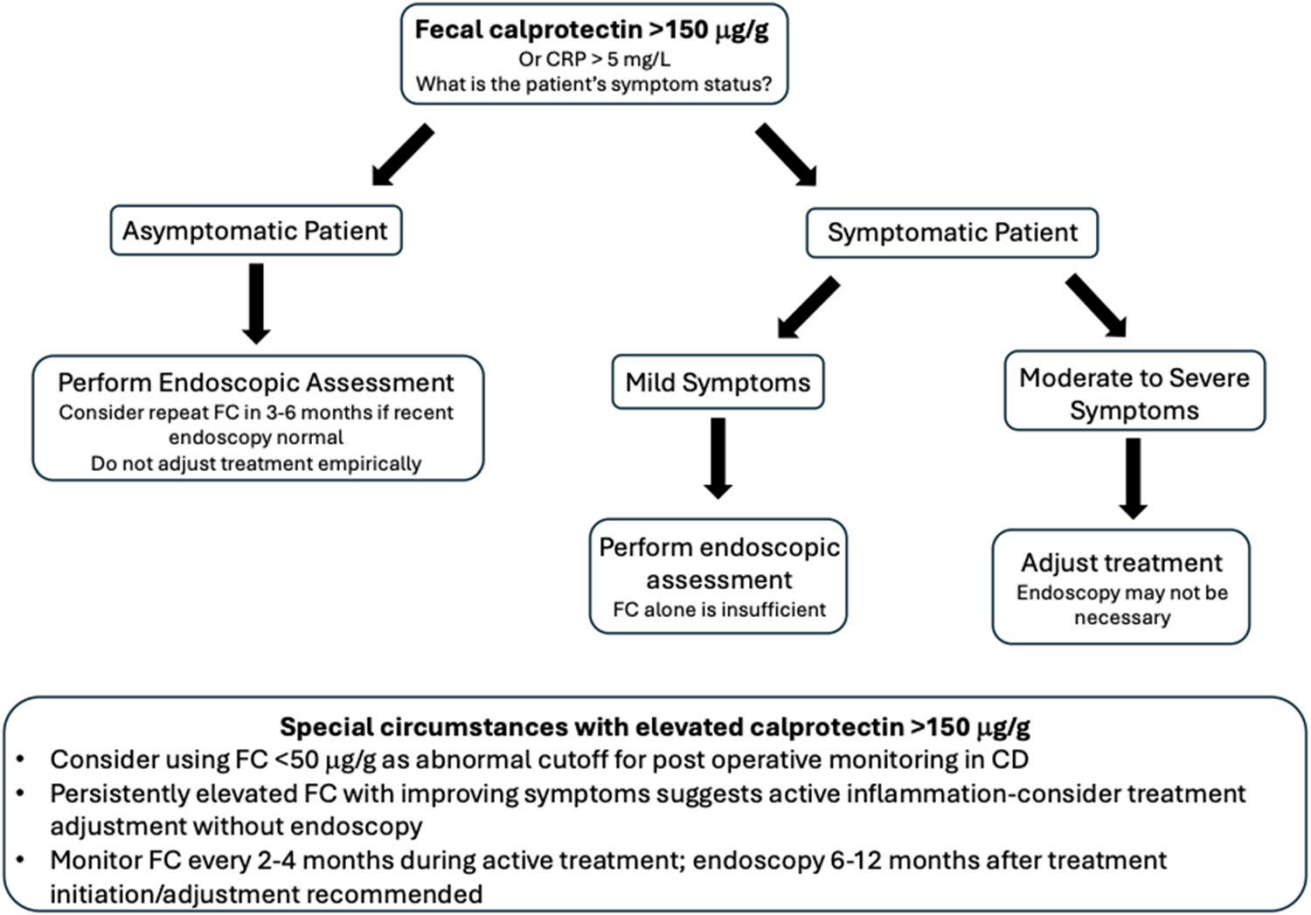
Ulcerative colitis and Crohn’s disease treatment recommendations in setting of elevated biomarkers based on clinical presentation. In patients with calprotectin over 150 μg/g or CRP > 5 mg/L, assess symptom status. Those who are asymptomatic may require endoscopic assessment for further evaluation or short interval repeat biomarkers. Those with mild symptoms typically require endoscopic assessment (see second bullet point in special circumstances). Those with moderate to severe symptoms, may undergo therapy adjustment (i.e. check drug levels and adjust) or endoscopic evaluation if options for changes in therapy are limited or endoscopy is needed for other reasons (i.e. rule out CMV). Pouchitis recommendations are discussed in the text. Abbreviations: CRP: C reactive protein, FC: fecal calprotectin, CMV: cytomegalovirus

Despite their widespread use and importance in a treat-to-target approach, there is considerable variability in the application of biomarkers in clinical practice. This guide aims to summarize evidence and recommendations for the use of biomarkers in common IBD scenarios, drawing from STRIDE II as well as the American Gastroenterological Association (AGA) guidelines for the use of biomarkers (see Fig. 1) [11, 12].

## Biomarker Characteristics

### Serum Biomarkers

CRP, an acute-phase reactant produced by the liver, can be indicative of any inflammatory process or systemic illness ranging from rheumatoid arthritis to pneumonia, and is thus not specific to IBD. Nonetheless, CRP is still widely utilized and possesses several characteristics valuable for disease monitoring. CRP has a short half-life of about 19 hours, making it a useful measure of disease response in the short term, including assessing response to steroids or infliximab in patients admitted with acute severe ulcerative colitis [13]. CRP is also important to prognosis in other settings: normalization in CRP at 8- and 14-weeks following treatment initiation has been associated with remission at 1 year [14].

CRP however has a relatively low sensitivity for endoscopic inflammation. For example, in those with UC, CRP only modestly correlates with endoscopic disease activity with a sensitivity of 51–53% and specificity of 69–71% [15]. While CRP likely performs better in CD, it is oftentimes still not elevated when only mild or moderate inflammation is present [16]. Additionally, there is significant inter-individual variability in CRP as 15% or more of patients with IBD, including those with certain genetic polymorphisms, do not have an elevated CRP, even when significant inflammation is present [17, 18]. Thus, while an important short-term target, CRP should not be used as a surrogate for healing. Clinicians should also be aware that units on CRP are variable (at times reported as mg/dL and other times mg/L).

Erythrocyte sedimentation rate (ESR) reflects the increase in plasma viscosity due to the presence of acute-phase reactant proteins during inflammation. Therefore, ESR is influenced by both anemia and polycythemia. ESR can additionally be influenced by certain physiologic states and is known to increase in older age and pregnancy, making this a somewhat less accurate, less widely used, and at times more difficult to interpret biomarker [13].

### Stool Biomarkers

Fecal calprotectin, a protein released by neutrophils involved in inflammation in the gastrointestinal tract, more closely reflects disease activity than CRP [19]. However, while more specific, FC may still be elevated in other scenarios which do not represent active IBD including infection, drug-induced enterocolitis (NSAIDs, immunotherapy, etc.), diverticulitis or even colorectal cancer [20, 21]. In patients with IBD where infection is common and may mimic a flare, it is important to simultaneously test for enteric pathogens which may be the cause of the elevated FC [22].

FC values may demonstrate significant variability, even in samples collected on the same day or using different assays on the same sample [23]. Fortunately, values are less variable at the lower end of the range, which is oftentimes where FC is most helpful [23, 24]. Some intra-individual variability may be reduced by collecting samples at the same time of day such as the first bowel movement in the morning [25]. There is no set definition for a normal FC. Often used values for a normal cutoff range from 50 to 250 μg/g with lower values correlating better with endoscopic remission (i.e. MES 0 or 1 for UC) [26]. While less invasive than tests such as a colonoscopy, there is lower patient adherence to stool tests as compared to serum and about one third of patients find the stool collection to be difficult [27]. As with other biomarkers, a baseline calprotectin is important to obtain to appropriately interpret subsequent results.

Like FC, fecal lactoferrin is produced by neutrophils at sites of inflammation in the gastrointestinal tract. Relatively limited data exists regarding the accuracy of lactoferrin but some studies have shown that, like FC, this may be superior to CRP [28].

## Crohn’s Disease

In CD, achieving early mucosal healing can prevent irreversible bowel damage, changing the course of the disease [6, 29]. Patient symptoms have not been shown to correlate well with disease activity and therefore cannot be used in isolation to assess disease status [30]. FC, especially in patients who have some degree of colon involvement (rather than ileum alone) has shown a stronger correlation with endoscopic activity than symptoms [31, 32]. A meta-analysis including over two thousand patients with CD showed a pooled sensitivity of 82% and specificity of 72% for FC with variable normal cutoffs utilized [19]. When used as part of the treat-to-target approach and checked 12 weeks after treatment initiation, FC has been shown to correlate with long term outcomes [33, 34]. Relapse can also be predicted by FC, with those with an elevated value having between a 53% and 83% probability of relapse in the subsequent two to three months [35]. In the case of CRP, levels < 5 mg/dl at week 14 in those treated with anti-tumor necrosis factor (anti-TNF) therapy have been associated with greater likelihood of sustained treatment response [36, 37]. Elevated CRP values at time of anti-TNF discontinuation are also associated with risk of relapse [38]. Using CRP in addition to FC has been shown to be superior to either biomarker alone [39].

### Symptomatic Remission

Currently, there are no randomized controlled trials comparing a symptom-based monitoring strategy to a biomarker-based strategy in patients with CD in symptomatic remission. In the 2023 AGA clinical practice guidelines, 12 cohort studies that included almost one thousand asymptomatic patients with CD were identified. In the approximately one third of those patients with elevated FC (variably defined as 200–300 μg/g), the risk of disease relapse was 4.8 times more likely as compared to those in symptomatic remission with a normal FC. Therefore, a conditional recommendation was made in favor of a biomarker-based strategy over symptom monitoring alone for those with CD in symptomatic remission, with consideration of biomarkers followed every 6–12 months. However, in those asymptomatic patients who prefer to avoid the burden of stool collection or the worry that false positives may bring, symptom-based monitoring can be a reasonable alternative [12].

If biomarkers are normal (i.e. FC less than 150 μg/g or CRP less than 5 mg/L) in asymptomatic patients and they have undergone a normal endoscopic evaluation within the past three years, it is unlikely that any significant inflammation is present. If biomarkers are elevated, endoscopic evaluation should typically be the next step with consideration for possible repeat of biomarkers within 3–6 months in certain low-risk groups (i.e. recent normal endoscopy) with minimally elevated biomarkers [12]. Radiologic evaluation may be an acceptable alternative.

### Mild Symptoms

In patients with symptomatic CD, a biomarker-based strategy is superior to symptoms alone in guiding treatment changes. In the CALM study, 244 symptomatic patients with CD were randomized to receive standard clinical management as compared to a “tight control” strategy that included treatment escalation based on FC > 250 μg/g or CRP greater than 5 mg/L. Statistically significantly more patients in the tight control group as compared to the standard of care group (23 vs 37%) achieved deep remission by 48 weeks [8].

One potential drawback to a biomarker-based strategy in this symptomatic group is the potential delay in treatment while awaiting results of biomarker testing, however up to 20% of patients with symptoms suggestive of active CD may be in endoscopic remission and treated inappropriately if decisions are made based on symptoms alone [40]. In those patients who have typical CD symptoms but normal biomarkers, endoscopy or abdominal imaging should be pursued as a next step to evaluate for inflammation.

For patients who are mildly symptomatic with elevated biomarkers, endoscopic evaluation should typically be pursued. Note however that in patients with improving symptoms with lack of normalization or persistently elevated biomarkers, a clinician should consider making lower risk treatment adjustments such as checking drug levels or dose escalating a therapy prior to endoscopy. Following changes in therapy, a treat-to-target approach should again then be utilized with subsequent repeat biomarkers and endoscopy.

### Moderate to Severe Symptoms

In patients with CD and moderate-to-severe symptoms with elevated biomarkers, changes in therapy should be made on a case-by-case basis, but endoscopy may not be necessary in this group with a high pretest probability for inflammation and elevated biomarkers. In patients with moderate-to-severe symptoms but normal biomarkers, endoscopic evaluation should be performed [12].

There is conflicting data on drug dosing changes based on biomarker and clinical response. The STARDUST trial did not show an increased rate of endoscopic improvement at week 48 in patients with moderate to severe disease on ustekinumab followed with a treat-to-target strategy using symptoms and biomarkers as compared to those who underwent therapy adjustment in response to symptoms. However, these results may not be generalizable to other patients with CD on different therapies or naïve to biologics [41].

### Postoperative Recurrence

For those patients with CD who have undergone surgically induced remission in the past year and are at low risk for disease recurrence or are on therapy to prevent recurrence, it may be reasonable to use FC to assess for recurrence as opposed to routine endoscopy; however in this case a lower FC threshold of 50 μg/g or less may be favored, especially in those not on therapy [12]. Any patient with a FC over this value should undergo endoscopic evaluation. For those who are at high risk for disease recurrence (younger age, smokers, history of multiple surgeries, penetrating disease phenotype, or long segment bowel resection) and are not on therapy, endoscopic evaluation should be performed within 6–12 months of surgically induced remission regardless of biomarkers [42–44]. CRP is likely not sufficiently sensitive to be used alone to assess for disease recurrence after surgically induced remission.

## Ulcerative Colitis

In UC, FC is much more sensitive than CRP and has become a cornerstone in disease monitoring [45]. In a prospective multicenter study of ulcerative colitis patients in clinical remission with FC measured every 4 weeks, elevations in FC were present up to 3 months prior to clinical symptoms, with two monthly measurements > 300 μg/g predicting a flare with 100% specificity [46]. A FC ≤ 168 μg/g has been shown to predict a sustained clinical response at one year with 83% sensitivity and 74% specificity [47]. FC may not however be sufficiently sensitive to differentiate mild endoscopic activity (MES 1) from endoscopic remission (MES 0), which has been associated with a lower risk of relapse [48]. FC may also be less accurate in proctitis as opposed to left sided or extensive colitis and may more closely correlate with extent of disease than disease severity [49, 50].

### Symptomatic Remission

For those patients with UC in symptomatic remission, the addition of routine biomarker monitoring may be an important method for predicting disease relapse. In AGA’s 2023 guidelines, 17 cohort studies with 1286 patients with UC in symptomatic remission were identified. Patients with an elevated FC (most often defined as > 150 μg/g) were 4.4 times as likely to have recurrent symptoms within one year as compared to those with normal FC [11].

Therefore, in this group of patients, consideration should be given to the routine assessment of biomarkers every 6–12 months depending on patient and provider preference. Certain drawbacks may exist for this approach: stool collection for FC is not without burden on patients, serum CRP may not be sufficiently sensitive, and an elevated biomarker may cause some distress, especially as 26.4% of patients in symptomatic remission with FC > 150 did not have significant disease activity. Due to the relatively high rate of false positives in the asymptomatic group, in the event of an elevated FC level, consideration can be made for endoscopic evaluation as the next step or, alternatively, to repeat biomarkers in 3–6 months [11].

A FC of < 150 μg/g in an asymptomatic patient with UC is strongly correlated with endoscopic improvement: only 4.3% of this group was misclassified as having endoscopic improvement (MES 0 or 1) when they had moderate or severe inflammation (MES 2 or 3) [11]. This high negative predictive value suggests that patients who prefer to avoid endoscopic procedures may reasonably opt to forgo colonoscopy in the final stage of a treat-to-target approach [11].

### Mild Symptoms

For those with symptoms, it may be reasonable to proceed directly to endoscopic assessment as a normal biomarker may not be sufficient to rule out active inflammation. A FC of 150 in this scenario had 14.5% false negatives and 15.5% false positives; CRP of < 5 mg/dl has 18.5% false negatives and 11.5% false positives [11]. Low risk changes such as adjustment of a biologic after reactive drug monitoring may be pursued prior to endoscopic evaluation.

### Moderate to Severe Symptoms

In a pooled analysis of 2586 patients in six clinical trials with UC treated with biologics or tofacitinib and moderate-to-severe *symptoms*, 10–15% of patients had only mildly active disease or disease in endoscopic remission [51]. Therefore, treatment decisions made on symptoms alone without the use of biomarkers may result in unnecessary corticosteroids or changes of maintenance therapy. In this scenario, FC can be a useful test with a false negative rate < 5% for FC < 150 μg/g in those with moderate-to-severe symptoms and may obviate the need for endoscopy for disease assessment alone [12]. In this case, steps should be taken to determine if a change or optimization in therapy is warranted such as evaluating drug levels or offering re-induction of small molecule.

### Pouchitis

While biomarker assessment in pouchitis has not been routinely recommended, there is a significant potential for use [52]. FC correlates with endoscopic and histologic disease activity in pouchitis, and, at times, elevations may precede the clinical diagnosis of pouchitis by as much as two months [53, 54]. Additionally, FC can be used to differentiate between inflammatory and non-inflammatory disorders of the pouch with levels below 100 μg/g ruling out pouchitis with high sensitivity [55].

## Managing Discordant Findings Between Symptoms and Biomarkers

Clinicians frequently encounter scenarios where clinical symptoms and biomarker results appear to be discordant. These situations require careful clinical judgement and take into account several patient specific factors, described in more detail above.

### Asymptomatic Patients with Elevated Biomarkers

This pattern may suggest subclinical inflammation and can be an early sign of disease relapse. For CD and UC patients in clinical remission with elevated FC (> 150 μg/g) or CRP (> 5 mg/L), options include endoscopic evaluation to assess mucosal inflammation or repeating biomarkers in 3–6 months, particularly in patients with recently confirmed endoscopic remission or those with a history of biomarker fluctuation without clinical change. Risk stratification based on disease history, previous biomarker patterns, and endoscopic findings can guide the descision.

### Symptomatic Patients with Normal Biomarkers

When patients report symptoms suggestive of active disease, but biomarkers remain normal, endoscopic evaluation is generally warranted. This is particularly important in patients with mild-to-moderate symptoms where the pre-test probability of active inflammation is intermediate. In patients with severe symptoms and normal biomarkers, prompt endoscopic assessment is essential to rule out active inflammation and consider alternative diagnoses such as irritable bowel syndrome, bile acid malabsorption, small intestinal bacterial overgrowth, or stricturing disease without active inflammation.

## Future Directions

Data supports the use of biomarkers in symptomatic patients and with changing therapies, however more research is needed regarding the role and timing of biomarker monitoring in the asymptomatic patient with IBD. Additionally, more research is needed to help determine the best biomarker thresholds which distinguish active IBD from inactive IBD in different clinical scenarios.

Biomarkers which can be measured at home or as point of care tests, such as wearable devices that can measure IL1β and CRP content in sweat are of great interest [56]. At home point of care fecal calprotectin test kits analyzed using a patient’s smartphone have been shown to correlate well with traditional ELISA testing at levels < 500 μg/g and may allow more frequent monitoring as well as improve access for those who live far from labs offering calprotectin testing [57]. Lastly, novel biomarkers including those that use transcriptomic and proteomic data have shown promise and may ultimately be more accurate in predicting treatment response and disease flares than traditional biomarkers [58].

## Conclusions

Resolution of symptoms is important to both patients and providers, but a treat-to-target approach using objective data to assess for response aims for deeper levels of remission that prevents disease progression and complications. While biomarkers cannot replace endoscopy, they have become a common part of clinical practice essential for disease treatment and monitoring.

## Key references

- Turner D, Ricciuto A, Lewis A, et al. STRIDE-II: An Update on the Selecting Therapeutic Targets in Inflammatory Bowel Disease (STRIDE) Initiative of the International Organization for the Study of IBD (IOIBD): Determining Therapeutic Goals for Treat-to-Target strategies in IBD. *Gastroenterology*. 2021;160(5):1570-1583. 10.1053/j.gastro.2020.12.031.
  - An evidence and consensus-based guide to a treat-to-target approach in managing IBD.
- Singh S, Ananthakrishnan AN, Nguyen NH, et al. AGA Clinical Practice Guideline on the Role of Biomarkers for the Management of Ulcerative Colitis. *Gastroenterology*. 2023;164(3):344–372. 10.1053/j.gastro.2022.12.007.
  - AGA guideline which outlines 7 conditional recommendations for biomarker use in ulcerative colitis created by a multidisciplinary panel of content experts and guideline methodologists.
- Ananthakrishnan AN, Adler J, Chachu KA, et al. AGA Clinical Practice Guideline on the Role of Biomarkers for the Management of Crohn’s Disease. *Gastroenterology*. 2023;165(6):1367-1399. 10.1053/j.gastro.2023.09.029.
  - AGA guideline which outlines 11 conditional recommendations for biomarker use in Crohn’s disease created by a multidisciplinary panel of content experts and guideline methodologists.

## Data Availability

No datasets were generated or analysed during the current study.

## References

[1] Gracie DJ, Williams CJM, Sood R, et al. Poor correlation between clinical disease activity and mucosal inflammation, and the role of psychological comorbidity, in inflammatory bowel disease. Am J Gastroenterol. 2016;111(4):541–51. 10.1038/ajg.2016.59. 27002800 10.1038/ajg.2016.59

[2] Peyrin-Biroulet L, Sandborn W, Sands BE, et al. Selecting therapeutic targets in inflammatory bowel disease (STRIDE): Determining therapeutic goals for treat-to-target. Am J Gastroenterol. 2015;110(9):1324–38. 10.1038/ajg.2015.233. 26303131 10.1038/ajg.2015.233

[3] Turner D, Ricciuto A, Lewis A, et al. STRIDE-II: An update on the selecting therapeutic targets in inflammatory bowel disease (STRIDE) initiative of the international organization for the study of IBD (IOIBD): Determining therapeutic goals for treat-to-target strategies in IBD. Gastroenterology. 2021;160(5):1570–83. 10.1053/j.gastro.2020.12.031. 33359090 10.1053/j.gastro.2020.12.031

[4] Schnitzler F, Fidder H, Ferrante M, et al. Mucosal healing predicts long-term outcome of maintenance therapy with infliximab in Crohnʼs disease. Inflamm Bowel Dis. 2009;15(9):1295–301. 10.1002/ibd.20927. 19340881 10.1002/ibd.20927

[5] Baert F, Moortgat L, Van Assche G, et al. Mucosal healing predicts sustained clinical remission in patients with early-stage Crohn’s disease. Gastroenterology. 2010;138(2):463–8. 10.1053/j.gastro.2009.09.056. 19818785 10.1053/j.gastro.2009.09.056

[6] Neurath MF, Travis SPL. Mucosal healing in inflammatory bowel diseases: a systematic review. Gut. 2012;61(11):1619–35. 10.1136/gutjnl-2012-302830. 22842618 10.1136/gutjnl-2012-302830

[7] Frøslie KF, Jahnsen J, Moum BA, Vatn MH. Mucosal healing in inflammatory bowel disease: Results from a Norwegian population-based cohort. Gastroenterology. 2007;133(2):412–22. 10.1053/j.gastro.2007.05.051. 17681162 10.1053/j.gastro.2007.05.051

[8] Colombel JF, Panaccione R, Bossuyt P, et al. Effect of tight control management on Crohn’s disease (CALM): a multicentre, randomised, controlled phase 3 trial. The Lancet. 2017;390(10114):2779–89. 10.1016/S0140-6736(17)32641-7. 10.1016/S0140-6736(17)32641-7 29096949

[9] Jones J, Loftus EV, Panaccione R, et al. Relationships between disease activity and serum and fecal biomarkers in patients with Crohn’s disease. Clin Gastroenterol Hepatol. 2008;6(11):1218–24. 10.1016/j.cgh.2008.06.010. 18799360 10.1016/j.cgh.2008.06.010

[10] Murthy SK, Feuerstein JD, Nguyen GC, Velayos FS. AGA clinical practice update on endoscopic surveillance and management of colorectal dysplasia in inflammatory bowel diseases: Expert review. Gastroenterology. 2021;161(3):1043-1051.e4. 10.1053/j.gastro.2021.05.063. 34416977 10.1053/j.gastro.2021.05.063

[11] Singh S, Ananthakrishnan AN, Nguyen NH, et al. AGA clinical practice guideline on the role of biomarkers for the management of ulcerative colitis. Gastroenterology. 2023;164(3):344–72. 10.1053/j.gastro.2022.12.007. 36822736 10.1053/j.gastro.2022.12.007

[12] Ananthakrishnan AN, Adler J, Chachu KA, et al. AGA clinical practice guideline on the role of biomarkers for the management of Crohn’s disease. Gastroenterology. 2023;165(6):1367–99. 10.1053/j.gastro.2023.09.029. 37981354 10.1053/j.gastro.2023.09.029

[13] Mendoza JL, Abreu MT. Biological markers in inflammatory bowel disease: Practical consideration for clinicians. Gastroenterol Clin Biol. 2009;33:S158–73. 10.1016/S0399-8320(09)73151-3. 20117339 10.1016/S0399-8320(09)73151-3

[14] Sollelis E, Quinard RM, Bouguen G, et al. Combined evaluation of biomarkers as predictor of maintained remission in Crohn’s disease. World J Gastroenterol. 2019;25(19):2354–64. 10.3748/wjg.v25.i19.2354. 31148906 10.3748/wjg.v25.i19.2354 PMC6529885

[15] Yoon JY, Park SJ, Hong SP, Kim TI, Kim WH, Cheon JH. Correlations of C-reactive protein levels and erythrocyte sedimentation rates with endoscopic activity indices in patients with ulcerative colitis. Dig Dis Sci. 2014;59(4):829–37. 10.1007/s10620-013-2907-3. 24352705 10.1007/s10620-013-2907-3

[16] Solem CA, Loftus EV, Tremaine WJ, Harmsen WS, Zinsmeister AR, Sandborn WJ. Correlation of C-Reactive protein with clinical, endoscopic, histologic, and radiographic activity in inflammatory bowel disease. Inflamm Bowel Dis. 2005;11(8):707–12. 10.1097/01.MIB.0000173271.18319.53. 16043984 10.1097/01.mib.0000173271.18319.53

[17] Moran CJ, Kaplan JL, Winter HS. Genetic variation affects C-Reactive protein elevations in Crohn’s disease. Inflamm Bowel Dis. 2018;24(9):2048–52. 10.1093/ibd/izy100. 29718222 10.1093/ibd/izy100

[18] Mosli MH, Zou G, Garg SK, et al. C-Reactive protein, fecal calprotectin, and stool lactoferrin for detection of endoscopic activity in symptomatic inflammatory bowel disease patients: A systematic review and meta-analysis. Am J Gastroenterol. 2015;110(6):802–19. 10.1038/ajg.2015.120. 25964225 10.1038/ajg.2015.120

[19] Rokkas T, Portincasa P, Koutroubakis IE. Fecal calprotectin in assessing inflammatory bowel disease endoscopic activity: a diagnostic accuracy meta-analysis. J Gastrointestin Liver Dis. 2018;27(3):299–306. 10.15403/jgld.2014.1121.273.pti. 30240474 10.15403/jgld.2014.1121.273.pti

[20] Pathirana WGW, Chubb SP, Gillett MJ, Vasikaran SD. Faecal calprotectin. Clin Biochem Rev. 2018;39(3):77–90. 30828114 PMC6370282

[21] von Roon AC, Karamountzos L, Purkayastha S, et al. Diagnostic precision of fecal calprotectin for inflammatory bowel disease and colorectal malignancy. Am J Gastroenterol. 2007;102(4):803–13. 10.1111/j.1572-0241.2007.01126.x. 17324124 10.1111/j.1572-0241.2007.01126.x

[22] Axelrad JE, Joelson A, Green PHR, et al. Enteric infections are common in patients with flares of inflammatory bowel disease. Am J Gastroenterol. 2018;113(10):1530–9. 10.1038/s41395-018-0211-8. 30072777 10.1038/s41395-018-0211-8 PMC7939066

[23] D’Amico F, Rubin DT, Kotze PG, et al. International consensus on methodological issues in standardization of fecal calprotectin measurement in inflammatory bowel diseases. United Eur Gastroenterol J. 2021;9(4):451–60. 10.1002/ueg2.12069. 10.1002/ueg2.12069 PMC8259254 33961734

[24] Kristensen V, Malmstrøm GH, Skar V, Røseth A, Moum B. Clinical importance of faecal calprotectin variability in inflammatory bowel disease: intra-individual variability and standardisation of sampling procedure. Scand J Gastroenterol. 2016;51(5):548–55. 10.3109/00365521.2015.1117650. 26634305 10.3109/00365521.2015.1117650

[25] Lasson A, Stotzer PO, Öhman L, Isaksson S, Sapnara M, Strid H. The intra-individual variability of faecal calprotectin: A prospective study in patients with active ulcerative colitis. J Crohns Colitis. Published online July 2014. 10.1016/j.crohns.2014.06.002. 10.1016/j.crohns.2014.06.002 25008478

[26] Patel A, Panchal H, Dubinsky MC. Fecal calprotectin levels predict histological healing in ulcerative colitis. Inflamm Bowel Dis. 2017;23(9):1600–4. 10.1097/MIB.0000000000001157. 28590341 10.1097/MIB.0000000000001157

[27] Khakoo NS, Lewis A, Roldan GA, et al. Patient adherence to fecal calprotectin testing is low compared to other commonly ordered tests in patients with inflammatory bowel disease. Crohns Colitis 360. 2021;3(3):otab028. 10.1093/crocol/otab028. 36776647 10.1093/crocol/otab028 PMC9802323

[28] Langhorst J, Elsenbruch S, Koelzer J, Rueffer A, Michalsen A, Dobos GJ. Noninvasive markers in the assessment of intestinal inflammation in inflammatory bowel diseases: Performance of fecal lactoferrin, calprotectin, and PMN-Elastase, CRP, and clinical indices. Am J Gastroenterol. 2008;103(1):162–9. 10.1111/j.1572-0241.2007.01556.x. 17916108 10.1111/j.1572-0241.2007.01556.x

[29] Ungaro RC, Yzet C, Bossuyt P, et al. Deep remission at 1 year prevents progression of early Crohn’s disease. Gastroenterology. 2020;159(1):139–47. 10.1053/j.gastro.2020.03.039. 32224129 10.1053/j.gastro.2020.03.039 PMC7751802

[30] Tse CS, Singh S, Valasek MA, et al. Prevalence and correlations of gastrointestinal symptoms with endoscopic and histologic mucosal healing in Crohn’s disease. Am J Gastroenterol. 2023;118(4):748–51. 10.14309/ajg.0000000000002122. 36623171 10.14309/ajg.0000000000002122

[31] Sipponen T, Savilahti E, Kolho KL, Nuutinen H, Turunen U, Färkkilä M. Crohnʼs disease activity assessed by fecal calprotectin and lactoferrin: Correlation with Crohnʼs disease activity index and endoscopic findings. Inflamm Bowel Dis. 2008;14(1):40–6. 10.1002/ibd.20312. 18022866 10.1002/ibd.20312

[32] D’Haens G, Ferrante M, Vermeire S, et al. Fecal calprotectin is a surrogate marker for endoscopic lesions in inflammatory bowel disease. Inflamm Bowel Dis. 2012;18(12):2218–24. 10.1002/ibd.22917. 22344983 10.1002/ibd.22917

[33] Lobatón T, López-García A, Rodríguez-Moranta F, Ruiz A, Rodríguez L, Guardiola J. A new rapid test for fecal calprotectin predicts endoscopic remission and postoperative recurrence in Crohn’s disease. J Crohns Colitis. 2013;7(12):e641–51. 10.1016/j.crohns.2013.05.005. 23810085 10.1016/j.crohns.2013.05.005

[34] Haisma SM, Verkade HJ, Scheenstra R, van der Doef HPJ, Bodewes FAJA, van Rheenen PF. Time-to-reach target calprotectin level in newly diagnosed patients with inflammatory bowel disease. J Pediatr Gastroenterol Nutr. 2019;69(4):466–73. 10.1097/MPG.0000000000002458. 31365486 10.1097/MPG.0000000000002458 PMC6750145

[35] Heida A, Park KT, van Rheenen PF. Clinical utility of fecal calprotectin monitoring in asymptomatic patients with inflammatory bowel disease. Inflamm Bowel Dis. 2017;23(6):894–902. 10.1097/MIB.0000000000001082. 28511198 10.1097/MIB.0000000000001082 PMC5434712

[36] Roblin X, Marotte H, Leclerc M, et al. Combination of C-reactive protein, infliximab trough levels, and stable but not transient antibodies to infliximab are associated with loss of response to infliximab in inflammatory bowel disease. J Crohns Colitis. 2015;9(7):525–31. 10.1093/ecco-jcc/jjv061. 25895875 10.1093/ecco-jcc/jjv061

[37] Hanauer SB, Feagan BG, Lichtenstein GR, et al. Maintenance infliximab for Crohn’s disease: the ACCENT I randomised trial. The Lancet. 2002;359(9317):1541–9. 10.1016/S0140-6736(02)08512-4. 10.1016/S0140-6736(02)08512-4 12047962

[38] Gisbert JP, Marín AC, Chaparro M. Systematic review: factors associated with relapse of inflammatory bowel disease after discontinuation of anti- TNF therapy. Aliment Pharmacol Ther. 2015;42(4):391–405. 10.1111/apt.13276. 26075832 10.1111/apt.13276

[39] Reinisch W, Panaccione R, Bossuyt P, et al. Biomarker correlation with endoscopic outcomes in patients with Crohn’s disease: data from CALM. J Crohns Colitis. 2018;12:S011–S011. 10.1093/ecco-jcc/jjx180.014.

[40] Peyrin-Biroulet L, Reinisch W, Colombel JF, et al. Clinical disease activity, C-reactive protein normalisation and mucosal healing in Crohn’s disease in the SONIC trial. Gut. 2014;63(1):88–95. 10.1136/gutjnl-2013-304984. 23974954 10.1136/gutjnl-2013-304984

[41] Danese S, Vermeire S, D’Haens G, et al. Treat to target versus standard of care for patients with Crohn’s disease treated with ustekinumab (STARDUST): an open-label, multicentre, randomised phase 3b trial. Lancet Gastroenterol Hepatol. 2022;7(4):294–306. 10.1016/S2468-1253(21)00474-X. 35120656 10.1016/S2468-1253(21)00474-X

[42] Regueiro M, Velayos F, Greer JB, et al. American gastroenterological association institute technical review on the management of Crohn’s disease after surgical resection. Gastroenterology. 2017;152(1):277-295.e3. 10.1053/j.gastro.2016.10.039. 27840073 10.1053/j.gastro.2016.10.039

[43] Lee KE, Cantrell S, Shen B, Faye AS. Post-operative prevention and monitoring of Crohn’s disease recurrence. Gastroenterol Rep (Oxf). 2022;10:goac070. 10.1093/gastro/goac070. 36405006 10.1093/gastro/goac070 PMC9667961

[44] Barnes EL, Lightner AL, Regueiro M. Perioperative and postoperative management of patients with Crohn’s disease and ulcerative colitis. Clin Gastroenterol Hepatol. 2020;18(6):1356–66. 10.1016/j.cgh.2019.09.040. 31589972 10.1016/j.cgh.2019.09.040

[45] Sands BE. Biomarkers of inflammation in inflammatory bowel disease. Gastroenterology. 2015;149(5):1275-1285.e2. 10.1053/j.gastro.2015.07.003. 26166315 10.1053/j.gastro.2015.07.003

[46] De VM, Louis EJ, Jahnsen J, et al. Consecutive fecal calprotectin measurements to predict relapse in patients with ulcerative colitis receiving infliximab maintenance therapy. Inflamm Bowel Dis. 2013;19(10):2111–7. 10.1097/MIB.0b013e31829b2a37. 23883959 10.1097/MIB.0b013e31829b2a37

[47] Guidi L, Marzo M, Andrisani G, et al. Faecal calprotectin assay after induction with anti-Tumour Necrosis Factor α agents in inflammatory bowel disease: Prediction of clinical response and mucosal healing at one year. Dig Liver Dis. 2014;46(11):974–9. 10.1016/j.dld.2014.07.013. 25096964 10.1016/j.dld.2014.07.013

[48] Yoon H, Jangi S, Dulai PS, et al. Incremental benefit of achieving endoscopic and histologic remission in patients with ulcerative colitis: A systematic review and meta-analysis. Gastroenterology. 2020;159(4):1262-1275.e7. 10.1053/j.gastro.2020.06.043. 32585306 10.1053/j.gastro.2020.06.043 PMC7658293

[49] Sakuraba A, Nemoto N, Hibi N, et al. Extent of disease affects the usefulness of fecal biomarkers in ulcerative colitis. BMC Gastroenterol. 2021;21(1):197. 10.1186/s12876-021-01788-4. 33933033 10.1186/s12876-021-01788-4 PMC8088576

[50] Kawashima K, Ishihara S, Yuki T, et al. Fecal calprotectin level correlated with both endoscopic severity and disease extent in ulcerative colitis. BMC Gastroenterol. 2016;16(1):47. 10.1186/s12876-016-0462-z. 27071448 10.1186/s12876-016-0462-z PMC4830074

[51] Dulai PS, Singh S, Jairath V, et al. Prevalence of endoscopic improvement and remission according to patient-reported outcomes in ulcerative colitis. Aliment Pharmacol Ther. 2020;51(4):435–45. 10.1111/apt.15577. 31755121 10.1111/apt.15577 PMC6989392

[52] Barnes EL, Agrawal M, Syal G, et al. AGA clinical practice guideline on the management of pouchitis and inflammatory pouch disorders. Gastroenterology. 2024;166(1):59–85. 10.1053/j.gastro.2023.10.015. 38128971 10.1053/j.gastro.2023.10.015 PMC11163976

[53] Ollech JE, Bannon L, Maharshak N, et al. Fecal calprotectin is increased in pouchitis and progressively increases with more severe endoscopic and histologic disease. Clin Gastroenterol Hepatol. 2022;20(8):1839-1846.e2. 10.1016/j.cgh.2021.11.012. 34798336 10.1016/j.cgh.2021.11.012

[54] Yamamoto T, Shimoyama T, Bamba T, Matsumoto K. Consecutive monitoring of fecal calprotectin and lactoferrin for the early diagnosis and prediction of pouchitis after restorative proctocolectomy for ulcerative colitis. Am J Gastroenterol. 2015;110(6):881–7. 10.1038/ajg.2015.129. 25916224 10.1038/ajg.2015.129

[55] Ardalan ZS, Friedman AB, Con D, et al. Accuracy of gastrointestinal ultrasound and calprotectin in the assessment of inflammation and its location in patients with an ileoanal pouch. J Crohns Colitis. 2022;16(1):79–90. 10.1093/ecco-jcc/jjab125. 34302729 10.1093/ecco-jcc/jjab125

[56] Jagannath B, Lin KC, Pali M, Sankhala D, Muthukumar S, Prasad S. A Sweat-based Wearable Enabling Technology for Real-time Monitoring of IL-1β and CRP as Potential Markers for Inflammatory Bowel Disease. Inflamm Bowel Dis. 2020;26(10):1533–42. 10.1093/ibd/izaa191. 32720974 10.1093/ibd/izaa191

[57] Heida A, Knol M, Kobold AM, Bootsman J, Dijkstra G, van Rheenen PF. Agreement between home-based measurement of stool calprotectin and ELISA results for monitoring inflammatory bowel disease activity. Clin Gastroenterol Hepatol. 2017;15(11):1742-1749.e2. 10.1016/j.cgh.2017.06.007. 28606846 10.1016/j.cgh.2017.06.007

[58] Argmann C, Hou R, Ungaro RC, et al. Biopsy and blood-based molecular biomarker of inflammation in IBD. Gut. 2023;72(7):1271–87. 10.1136/gutjnl-2021-326451. 36109152 10.1136/gutjnl-2021-326451 PMC10014487

